# Isolation, characterization, and biological evaluation of a potent anti-malarial drimane sesquiterpene from *Warburgia salutaris* stem bark

**DOI:** 10.1186/s12936-018-2439-6

**Published:** 2018-08-15

**Authors:** Zoxolo N. Nyaba, Pretty Murambiwa, Andy R. Opoku, Samson Mukaratirwa, Francis O. Shode, Mthokozisi B. C. Simelane

**Affiliations:** 1 School of Life Sciences, University of KwaZulu-Natal, Scottsville, Pietermaritzburg, 3209 South Africa; 2 School of Life Sciences, University of KwaZulu-Natal, Durban, 4041 South Africa; 3 Department of Biochemistry & Microbiology, University of Zululand, Private Bag X1001, KwaDlangezwa, 3886 South Africa; 4 Department of Biotechnology and Food Technology, Durban University of Technology, P O Box 1334, Durban, 4000 South Africa

**Keywords:** *Plasmodium falciparum*, Anti-malarial agents, *Warburgia salutaris*, Iso-mukaadial acetate, *Plasmodium berghei*, Sprague–Dawley rats

## Abstract

**Background:**

Malaria continues to be a global burden as the efficacy of most commercial anti-malarial drugs has been compromised by the evolution of parasite resistance. With the urgent need created for the development of alternative and more efficient anti-malarial drugs, this study focused on the evaluation of anti-malarial agents of the *Warburgia salutaris* stem bark.

**Methods:**

The stem bark was extracted with dichloromethane followed by silica gel column chromatography that led to the isolation of iso-mukaadial acetate, a drimanoid sesquiterpene. This compound was characterized by spectroscopic analysis (^1^H NMR, ^13^C NMR, IR and MS), and its structure was confirmed by X-ray crystallography. In vitro anti-plasmodial activity was investigated using a chloroquine sensitive NF54 *Plasmodium falciparum* strain. Cytotoxicity was measured using the MTT assay on HEK239 and HEPG2 cell lines. Chloroquine-sensitive *Plasmodium berghei* was used to infect Sprague–Dawley rats for in vivo studies. The *W. salutaris* crude extract and iso-mukaadial acetate were administered orally at 0.5; 1.5, 2.5 and 5 mg/kg, chloroquine was used as the reference drug. Determination of percentage parasitaemia, haematological parameters, and rat body weights was done throughout the experimental study period.

**Results:**

The crude extract and iso-mukaadial acetate showed very good activity on the inhibition of parasite growth (IC_50_ 0.01 ± 0.30 µg/ml) and (IC_50_ 0.44 ± 0.10 µg/ml), respectively, with iso-mukaadial acetate having cytotoxicity activity of 36.7 ± 0.8 and 119.2 ± 8.8 (µg/ml) on HEK293 and HEPG2 cells, respectively. The crude extract and iso-mukaadial acetate reduced percentage parasitaemia in a dose-dependent manner in comparison to the control. There were no significant differences in the haematological parameters in all the experimental groups in comparison to control group. This study proves that *W. salutaris* contains components (including iso-mukaadial acetate) that exhibit anti-malarial activity. This study scientifically validates the use of this plant in folk medicine.

**Conclusions:**

This study proves that Warburgia salutaris contains components (including iso-mukaadial acetate) that exhibit anti-malarial activity and scientifically validates the use of this plant in folk medicine.

## Background

Approximately 80% of rural African population is mainly dependent on traditional medicine to treat diseases [1], including malaria [2]. Historically, most anti-malarial drugs have been derived from plants [3]. UNESCO encourages research into local medicinal plants with the hope of finding anti-malarial drugs that are more efficient and could combat the artemisinin resistant malaria parasite [4]. Currently artemisinin-based combination therapy is recommended for treatment of falciparum malaria and its effectiveness against other plasmodial species [5]. In 1996 among the top 20 prescription dispensed, six were derived from natural products and the clinical use of drugs, such as artemisinin and taxol, further proved that plants are sources of novel drugs entities [6].

*Warburgia salutaris* (pepper-bark tree) is an evergreen medium-sized tree that can grow up to 10 metres in height. It is found in the southern African region, where it is used in traditional medicine. Most traditional concoctions are prepared from the inner bark of this tree [7]. Tablet forms of *W. salutaris* are available in the market for the treatment of bronchitis, chest infections and ulcers [8]; the leaf tablet form is a natural antibiotic, effective against oesophageal and oral thrush [8, 9]. Traditional use of the roots, bark and leaves is common in the treatment of venereal diseases, influenza, abdominal pain, ulcers, respiratory complaints and malaria [10]. There has been no scientific proof of this plant’s anti-malarial activity to date.

Some drimane sesquiterpenes have been isolated from this plant; these include: warburganal [11], found to possess, molluscicidal [12], antifungal and antibacterial [7, 13] activity; polygodial [14], with antifungal and antibacterial [7] activity; salutarisolide [15–17], muzigadial [18], possess antimicrobial and antifungal [7, 19]; ugandensidial, isopolygodial and mukaadial [20]; 11a-hydroxycinnamosmolide with anti-mycobacterial activity [21]. The primary aim for this study is to scientifically validate the use of *W. salutaris* for treating malaria in Zulu traditional medicine. An attempt was made to identify the active constituent in the plant extract. Animal studies were also carried out to determine the plant’s targets in parasite progression.

## Methods

### Collection and preparation of plant material

*Warburgia salutaris* stem bark was collected from the Botanical gardens of the University of KwaZulu-Natal, Pietermaritzburg Campus, KwaZulu-Natal, South Africa (29°37′30′′S, 30°24′14′′E). The plant was identified by the horticulturist and a voucher specimen was prepared (voucher number: NU0043932) and deposited at the University of KwaZulu-Natal, Pietermaritzburg herbarium. The bark was sun-dried for 5 days and crushed into a fine powder.

### Extraction and isolation

The fine stem bark powder (1 kg) was subjected to maceration with dichloromethane (1:5 w/v) for 3 days with periodic shaking. The maceration extract was filtered through Whatman (No. 1) filter paper and concentrated under reduced pressure at 45 °C. Silica gel column chromatography (60 × 1000 mm; Merck silica gel, 60: 0.063–0.200 mm) was used to isolate the compound from the DCM extract. The column was eluted with hexane: ethyl acetate gradient and the ratio 8:2 yielded 35 fractions. The fractions were grouped according to the thin layer chromatography (silica gel 60 aluminium sheets, F 254—Merck, Whitehouse Station, New Jersey, USA) profiles, into seven combined fractions (Cfrs). The spots were fixed on the TLC plates with a 20% H_2_SO_4_ in methanol mixture and viewed under UV light (254 nm). The combined fractions were then dried under a fume-hood overnight; Cfrs 2–4 produced a white amorphous powder (NN-01). NN-01 was recrystallized from methanol to give white needles (mpt. 95–98 °C); [α]_D_ = − 11(0.1M in CHCl_3_); HREI-MS: MF = C_17_H_24_O_5_, [M+Na]^+^ ion at m/z 331.1619 (calcd: 331.1521).

### Structural elucidation

**Fig. 1:**
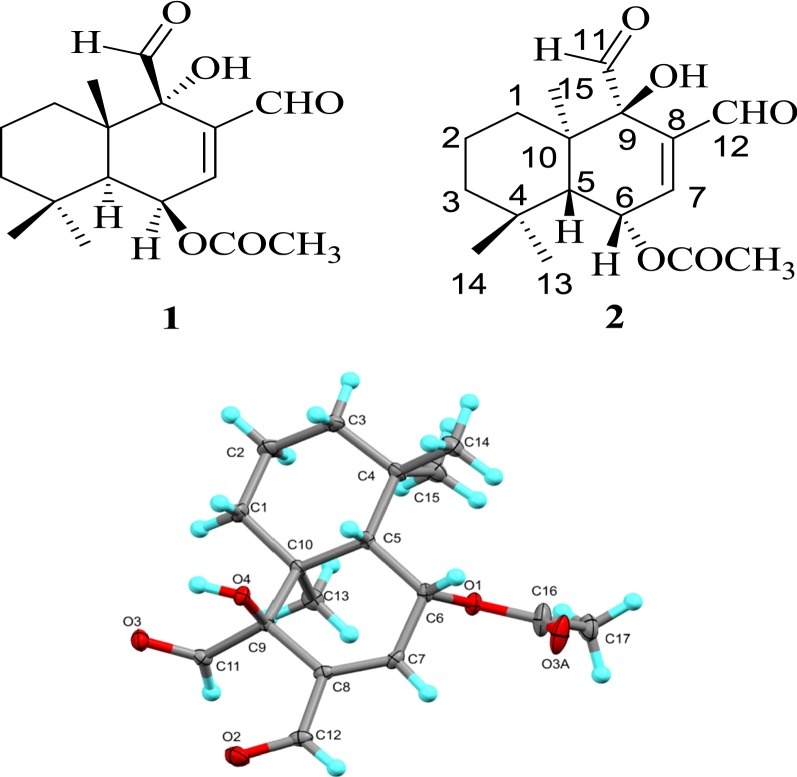
Single-crystal X-ray structure of iso-mukaadial acetate

The structure of NN-01 was determined using combined spectroscopic methods (FT-IR, Nuclear Magnetic Resonance (NMR) (^1^H, ^13^C, COSY, DEPT, NOESY, HMBC, and HSQC, and HREI-MS). Its FT-IR spectrum contained characteristic functional group frequencies: IR ν max (3450, 2942, 2848, 1743, 1717, 1691 cm^−1^). Its spectroscopic data (Table 1) are very similar to those of cinnamodial (**1**) [9]. However, its melting point (95–98 °C) was different from the melting point of cinnamodial (**1**) (135–137 °C) [9]. This difference in melting point led to the X-ray crystallographic study of NN-01 which confirmed its structure as iso-mukaadial acetate (**2**) (see Fig. 1).

**Table 1:** ^1^H- and ^13^C-NMR chemical shifts (δ, ppm) of cinnamodial (1) [7] and iso-mukaadial acetate (2)

Position	δc (ppm) (multiplicity)	δ_H_ (ppm)	δc (ppm) (multiplicity)	δ_H_ (ppm)
	Cinnamodial (1)		Iso-mukaadial acetate (2)	
1	31.9 (CH_2_)	1.77–1.39 (6H, *m*)	31.81 (CH_2_)	1.85–1.30 (6H, *m*)
2	19.9 (CH_2_)		19.93 (CH_2_)	
3	44.2 (CH_2_)		44.02 (CH_2_)	
4	34.0 (C)	–	34.0 (C)	–
5	45.2 (CH)	2.05 (1H, *d*, *J* = 4.6 Hz)	44.97 (CH)	2.08 (1H, *d*, *J* = 4.8 Hz)
6	66.2 (CH)	5.87 (1H, *t*, *J* = 4.6 Hz)	66.13 (CH)	5.91 (1H, *t*, *J* = 4.8 Hz)
7	148.5 (CH)	7.04 (1H, *d*, *J* = 4.6 Hz)	148.60 (CH)	7.01 (1H, *d*, *J* = 4.8 Hz)
8	141.3 (C)	–	141.0 (C)	–
9	77.5 (C)	–	77.2 (C)	–
9-OH	–	4.01 (1H, br *s*)		4.07 (1H, br *s*)
10	41.7 (C)		41.66 (C)	
11	201.0 (CH)	9.75 (1H, *s*)	201.44 (CH)	9.76 (1H, *s*)
12	192.9 (CH)	9.47 (1H, *s*)	193.01 (CH)	9.48 (1H, *s*)
13	32.6 (CH_3_)	1.02 (3H, *s*)	32.02 (CH_3_)	1.03 (3H, *s*)
14	21.3 (CH_3_)	1.16 (3H, *s*)	21.4 (CH_3_)	1.17 (3H, *s*)
15	17.7 (CH_3_)	1.34 (3H, *s*)	17.68 (CH_3_)	1.34 (3H, *s*)
OCOCH_3_	170.0 (C)		170.01 (C)	
OCOCH_3_	24.70 (CH_3_)	2.14 (3H, *s*)	24.75 (CH_3_)	2.17 (3H, *s*)

### MTT assay

The HepG2 (liver hepatocellular carcinoma) cells were obtained from the Highveld Biologicals (Pty) Ltd, Kelvin, SA, while the HEK293 (Human embryonic kidney) cells were provided by the University of the Witwatersrand, Medical school, Antiviral Gene Therapy Unit, SA. The modified method of Mosman [22] was used. The MTT assay is used to measure the cells metabolic activity, by reducing MTT (3-(4,5-dimethylthiazol-2-yl)-2,5-diphenyltetrazolium bromide) into formazan salt using the enzyme succinate-tetrazolium reductase within the cell. The cells were developed to semi-confluency, in flasks for tissue culture (25 cm^2^), in Eagle’s minimal essential medium containing, 100 U/ml penicillin; 100 μg/ml streptomycin as well as 10% fetal bovine serum. Cells were then seeded at a density of 21 × 10^3^ cells in each well, with medium (100 µl) in a 96 well plate. A 24 h incubation period (37 °C; 5% CO_2_) followed, after which fresh medium (100 µl) was added upon the removal of the old medium. Varying concentrations (50 µg/ml, 100 µg/ml and 150 µg/ml) of the compound were added in triplicates onto the cells then further incubated for 48 h at 37 °C. Cells without the experimental compound were used as the positive controls (100% viability).

The used medium was removed, after 48 h of incubation and, 100 µl new medium and 5 mg/ml MTT in PBS added, and this went into incubation (37 °C) for another 4 h. MTT and medium were then discarded, and 200 µl of DMSO was added per well, for dissolving the formazan. Absorbance of resulting mixture was then read with a Mindray 96A microplate reader (Vacutec, Hamburg, Germany) using a wavelength of 570 nm (detection λ) and that of 630 nm as the reference wavelength of nonspecific signals. The percentage viability of the cell was linked to absorbance and worked out in reference to the positive control using the equation: [(OD_570_ Treated)/(Control)]× 100. All experimentations were done in triplicates.

### In vitro anti-plasmodial activity

The compound was tested against a NF54 *Plasmodium falciparum* strain (chloroquine sensitive); the tests were done in triplicate. Continuous in vitro cultures of asexual erythrocytes stage of the parasite were maintained following the modified method of Trager and Jensen [23]. The viability of the parasite was evaluated by looking at the parasite lactate dehydrogenase activity, which was conducted following a modified method described by Makler et al. [24]. Test sample was made to a stock solution of 20 mg/ml in DMSO (100%). The stock solution was preserved at − 20 °C. Dilutions of the stock were made on the day of experimentation. Artesunate and chloroquine were the drugs of reference in the experiment.

Full dose–response was conducted on the test compound to find concentrations at which 50% of parasitic growth is inhibited (IC_50_ value). Primary compound concentration for the trial was at 100 μg/ml, thereafter successively diluted to concentration twice as much in medium to produce ten measures with varying concentrations; minimum concentration being 0.2 μg/ml. Chloroquine initial concentration was at 1000 ng/ml. Solvent concentrations in which the infected red blood cells were subjected to, had no significant consequence on the viability of parasite. A non-linear dose–response curve fitting analysis was used to calculate the IC_50_ values, with the version4.0 Graph Pad Prism software (Graph Pad Prism, Inc: San Diego, CA, USA, 1994–2003).

### In vivo antiplasmodial activity

#### Animals used

Male Sprague–Dawley rats were used, 16–18 weeks old, from the University of KwaZulu-Natal Biomedical research unit. The housing and feeding conditions were maintained as per recommended standards.

#### Parasite inoculation

Chloroquine sensitive *Plasmodium berghei* was obtained from the University of Cape Town, Clinical Pharmacology Division, South Africa. The parasite (10^5^ *P. berghei*) was introduced to the animals via an intraperitoneal injection.

#### Curative test

Male Sprague–Dawley rats (n = 48), were randomly grouped into non-*P. berghei* infected (n = 12) and *P. berghei* infected (n = 36) groups. Malaria induction was done via intra-peritoneal injection of 105 *P. berghei* strain ANKA parasitized RBCs. Following malaria induction, treatment was commenced after a 7 days pre-treatment period. The non-*P. berghei* infected experimental animals were further sub divided into two separate groups (n = 6/group), receiving oral dosing of 1.5 mg/kg crude extract and 1.5 mg/kg Iso-mukaadial acetate each. The *P. berghei* infected experimental animals were further sub divided into six separate experimental groups (n = 6/group): absolute control/not treated—vehicle group; positive control group-CQ 60 mg/kg (day 8–11); crude extract x5#—1.5 mg/kg (day 8–12); M. acetatex5#—1.5 mg/kg (day 8–12); Crude extract x1*—1.5 mg/kg (day 8); M. acetatex1*—1.5 mg/kg (day 8). The experimental animals were then monitored throughout the duration of the experimental period (21 days). *x1—treated for 1 day; #x5—treated for 5 days.

#### Determination of level of parasitaemia

Percentage parasitaemia was determined by thin blood smears prepared from the tail blood of each rat, fixed with methanol and stained with 20% Giemsa every day from day 3 to day 21. The animals were sacrificed on day 21 and determination of survival and haematology profiles were done.

Percentage parasitaemia levels were determined by counting the number of infected erythrocytes out of a hundred red blood cells in five random fields of the microscope. Average percent parasitaemia was calculated by using the formula below.

$$\% {\text{ Parasitaemia}} = \frac{\text{Number of parasitized RBC}}{\text{Total number of RBC count }} \times 100$$

#### Determination of body weight

Animal body weights were monitored to determine whether iso-mukaadial acetate and *W. salutaris* crude extract prevents weight loss that is commonly observed with increasing parasitaemia. Weights were taken at 3-day intervals until day 21. The rat grimace scale [25], from the National Centre for the Replacement Refinement and Reduction of Animals in Research (NC3Rs), was used to assess whether the animals were suffering during the experiment. Orbital tightening; nose and cheek flattening; ear change; and whisker change was used as parameters for the grimace scale.

#### Statistical analysis

In vitro data was analysed using one-way analysis of variance (ANOVA), the haematology data was analysed using group statistics, followed by Bonferroni post hoc test and parasitaemia and weight data was analysed using two-way analysis of variance. The mean ± SEM was calculated. The 2010 Microsoft Excel Program and version. 4.0 Graph Pad Prism software (Graph Pad Prism, Inc: San Diego, CA, USA, 1994–2003) were used for the statistical analysis of differences amongst the mean values calculated for the different trial groups, for IC_50_. *P* values ≤ 0.05 were taken as significant.

## Results and discussion

### In vitro antiplasmodial and cytotoxic activity

Many malaria-endemic areas are developing countries with infected people who cannot afford anti-malarial drugs and, therefore, rely on traditional medicine and medicinal plants as primary health care for the treatment of malaria [2]. Discovering novel phytochemical compounds for use in drug development, is the main goal of ethno-pharmacology [26]. Bioassay guided separation of the crude extract led to the isolation, purification and characterization of iso-mukaadial acetate. It was the first time that this compound was isolated from this plant, and the first time that any anti-malarial activity is being reported. The anti-malarial and cytotoxic activity results are depicted in Table 2.

**Table 2:** In vitro anti-plasmodial and cytotoxic activity of Iso-mukaadial acetate (NN-01)

Sample code	^a^IC_50_ (µg/ml)	CC_50_ (µg/ml)		Selectivity index	
		HEK293	HEPG2	HEK293	HEPG2
*W. Salutaris*	0.01 ± 0.30	21.30 ± 01	55.21 ± 11	2130	5521
NN-01	0.44 ± 0.10	36.7 ± 08	119.2 ± 88	83	270
CQ	4.9 ± 0.07 ng/ml	232.45 ± 21	350.04 ± 90	47,438	71,436
Art	< 2 ng/ml	N/D	N/D	N/D	N/D

*ND* Not determined

^a^IC_50_: Inhibitory concentration

In drug discovery, a compound is considered a success if it has an IC_50_ ≤ 1 µg/ml and a selectivity index that is at least ten times more active on the parasite than it is on human cell-lines [27]. Iso-mukaadial acetate shows good in vitro anti-malarial activity. There has been no report of any other isolated anti-malarial compound from *W. salutaris* in literature. Katiyar et al. suggests that natural sources have a potential of delivering natural compounds as base for synthetic analogues in drug discovery [26]. It is noteworthy to state that the high antiplasmodial activity of iso-mukaadial acetate makes it eligible for structural modification studies to reduce cytotoxicity while retaining or improving antiplasmodial activity.

### In vivo antiplasmodial results

**Fig. 2:**
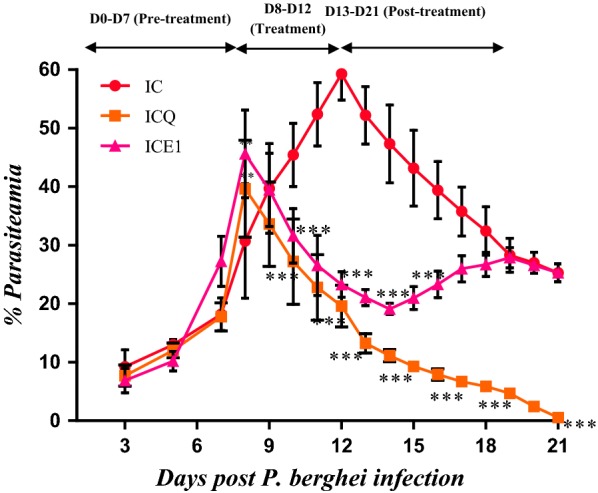
Percentage parasitaemia of *P. berghei* infected SD rats treated once with *Warburgia salutaris* crude extract (1.5 mg/kg) and CHQ (60 mg/kg). Values are presented as mean ± SEM. IC-infected without treatment; ICQ-infected chloroquine treated; ICE1-infected crude extract 1-day treatment. Two-way analysis of variance was carried out for all percentage parasitaemia data. Levels of significance, *P < 0.05, **P < 0.01, ***P < 0.001 in comparison to control group

**Fig. 3:**
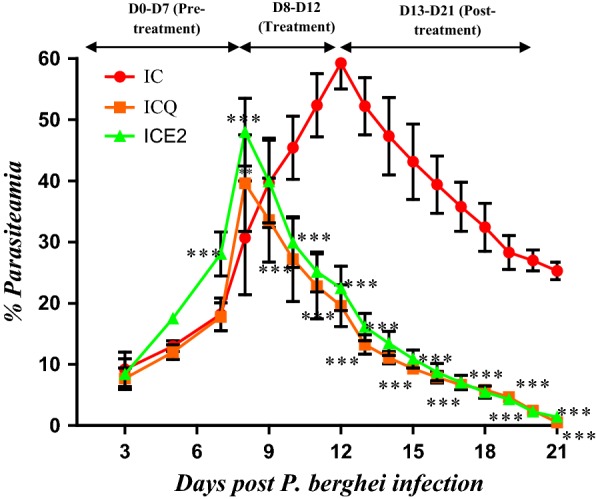
Percentage parasitaemia of *P. berghei* infected SD rats treated once daily (5-days) *W. salutaris* crude extract (1.5 mg/kg) and CHQ (60 mg/kg). Values are presented as mean ± SEM. IC-infected without treatment, ICQ-infected chloroquine treated, ICE2-infected, and crude extract 5-day treatment. Two-way analysis of variance was carried out for all percentage parasiteamia data. Levels of significance, *P < 0.05, **P < 0.01, ***P < 0.001 in comparison to control group

**Fig. 4:**
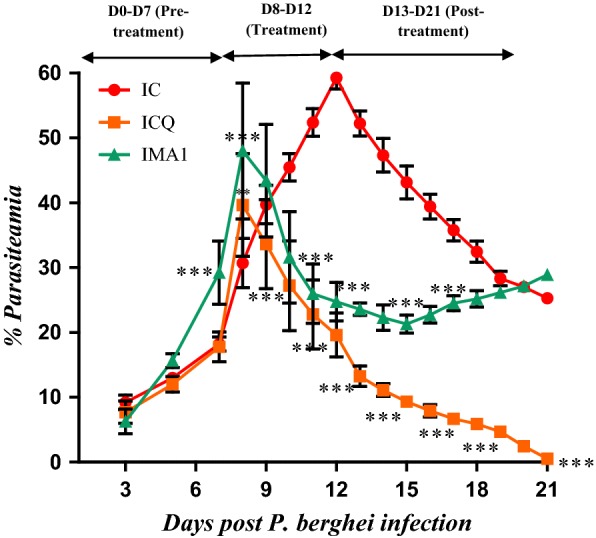
Percentage parasitaemia of *P. berghei* infected SD rats treated once with iso-mukaadial acetate (1.5 mg/kg) and CHQ (60 mg/kg). Values are presented as mean ± SEM. IC-infected without treatment; ICQ-infected chloroquine treated; IMA1-infected M. acetate 1-day treatment. Two-way analysis of variance was carried out for all percentage parasitaemia data. Levels of significance, *P < 0.05, **P < 0.01, ***P < 0.001 in comparison to control group

**Fig. 5:**
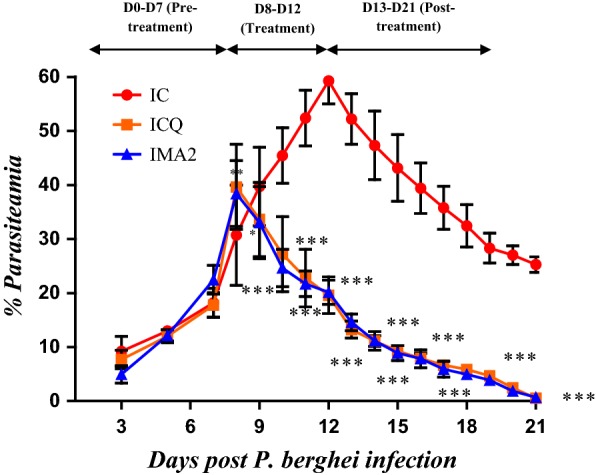
Percentage parasitaemia of *P. berghei* infected SD rats treated once daily (5 days) with iso-mukaadial acetate (1.5 mg/kg) and CHQ (60 mg/kg). Values are presented as mean ± SEM. IC-infected without treatment; ICQ-infected chloroquine treated; IMA2-infected iso-mukaadial acetate 5-day treatment. Two-way analysis of variance was carried out for all percentage parasitaemia data. Levels of significance, *P < 0.05, **P < 0.01, ***P < 0.001 in comparison to control group

*Plasmodium berghei* has been proven to be most effective in early investigation of anti-malarial activity for new test compounds [28]. Compounds with more than 30% parasite inhibition and that increase the rodents’ survival time [29], when compared to the absolute control are considered effective in standard screening tests. The study results showed varied antiplasmodial activity with the 5-day treatment groups showing optimum parasite inhibition, for both the crude extract and iso-mukaadial acetate, than those of the 1-day treatment. The results are depicted in Figs. 2, 3, 4, 5.

### Effects of *Warburgia salutaris* and iso-mukaadial acetate on haematology profile

The reduce red blood cell count and haematocrit in the study is consistent with previous malaria studies of severe malarial anaemia. A study showed that red blood cell destruction and decrease in red blood cell production in response to parasite invasion may lead to reduced haematocrit and haemoglobin levels in the blood of infected animals [30]. However, mechanisms by which haematology parameters were affected are not described in this study. Further plasma and serum studies may provide mechanisms by which observed malaria-induced haematology changes occurred. The study groups are represented as, UMA-Uninfected iso-mukaadial acetate treated; UCE-Uninfected crude extract treated; IC-infected without treatment; ICQ-infected chloroquine treated; ICE1-infected crude extract 1-day treatment; ICE2-infected crude extract 5-day treatment; IMA1-infected iso-mukaadial acetate 1-day treatment; IMA2-infected iso-mukaadial acetate 5-day treatment. Column statistics was carried out for all haematology data.

### Effects of *Warburgia salutaris* and iso-mukaadial acetate on animal weight

**Fig. 6:**
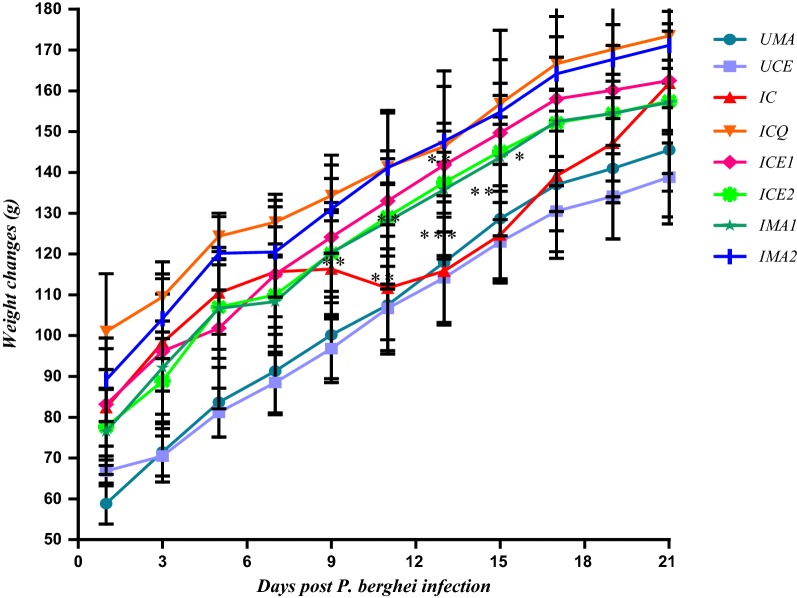
Difference in weight of *P. berghei* infected SD rats, treated with *W. salutaris* and iso-mukaadial acetate over a 21-day period. Values are presented as mean ± SEM. UMA-Uninfected M. acetate treated; UCE-Uninfected crude extract treated; IC-infected without treatment; ICQ-infected chloroquine treated; ICE1-infected crude extract 1-day treatment; ICE2-infected crude extract 5-day treatment; IMA1-infected iso-mukaadial acetate 1-day treatment; IMA2-infected iso-mukaadial acetate 5-day treatment. Two-way analysis of variance was carried out for all percentage parasitaemia data. Levels of significance, *P < 0.05, **P < 0.01, ***P < 0.001 in comparison to control group

The difference in weight was measured at 2-day intervals to determine whether the *W. salutaris* crude extract and iso-mukaadial acetate prevent weight loss, commonly observed in malaria. Effective anti-plasmodial products are expected to have protective effects against the loss of weight commonly observed in malaria infection, in this study, these protective effects were observed in the iso-mukaadial acetate and *W. salutaris* groups as shown in Fig. 6.

## Conclusion

The aim of this study was to scientifically validate the use of *W. salutaris* in Zulu traditional medicine. Results obtained show that *W. salutaris* possess anti-malarial activity, the active compound found to be iso-mukaadial acetate, which shows appreciable inhibition on parasite growth. The in vivo antiplasmodial studies with chloroquine-sensitive *P. berghei* show that *W. salutaris* has very good anti-malarial activity and protective effects on the survival of malaria-infected rodents. Iso-mukaadial acetate could serve as a lead compound for the development of a relatively potent drug in the treatment of malaria. However, more studies are required to determine the compounds mechanism of action on other in vitro models and on in vivo models as well. The results obtained support the use of *W. salutaris* in traditional medicine, to treat malaria.
